# Treatment Strategies for Hepatocellular Carcinoma—A Multidisciplinary Approach

**DOI:** 10.3390/ijms20061465

**Published:** 2019-03-22

**Authors:** Isabella Lurje, Zoltan Czigany, Jan Bednarsch, Christoph Roderburg, Peter Isfort, Ulf Peter Neumann, Georg Lurje

**Affiliations:** 1 Department of Surgery and Transplantation, University Hospital RWTH Aachen, 52074 Aachen, Germany; ibarth@ukaachen.de (I.L.); zczigany@ukaachen.de (Z.C.); jbednarsch@ukaachen.de (J.B.); uneumann@ukaachen.de (U.P.N.); 2 Department of Internal Medicine III, University Hospital RWTH Aachen, 52074 Aachen, Germany; croderburg@ukaachen.de; 3 Department of Gastroenterology/Hepatology, Campus Virchow Klinikum and Charité Mitte, Charité University Medicine Berlin, 13353 Berlin, Germany; 4 Department for Diagnostic and Interventional Radiology, University Hospital RWTH Aachen, 52074 Aachen, Germany; isfort@ukaachen.de; 5 Department of Surgery, Maastricht University Medical Centre (MUMC), 6229 ET Maastricht, The Netherlands

**Keywords:** Hepatocellular carcinoma, liver cirrhosis, liver transplantation, liver resection, multimodal treatment

## Abstract

Hepatocellular carcinoma (HCC) is the most common primary tumor of the liver and its mortality is third among all solid tumors, behind carcinomas of the lung and the colon. Despite continuous advancements in the management of this disease, the prognosis for HCC remains inferior compared to other tumor entities. While orthotopic liver transplantation (OLT) and surgical resection are the only two curative treatment options, OLT remains the best treatment strategy as it not only removes the tumor but cures the underlying liver disease. As the applicability of OLT is nowadays limited by organ shortage, major liver resections—even in patients with underlying chronic liver disease—are adopted increasingly into clinical practice. Against the background of the oftentimes present chronical liver disease, locoregional therapies have also gained increasing significance. These strategies range from radiofrequency ablation and trans-arterial chemoembolization to selective internal radiation therapy and are employed in both curative and palliative intent, individually, as a bridging to transplant or in combination with liver resection. The choice of the appropriate treatment, or combination of treatments, should consider the tumor stage, the function of the remaining liver parenchyma, the future liver remnant volume and the patient’s general condition. This review aims to address the topic of multimodal treatment strategies in HCC, highlighting a multidisciplinary treatment approach to further improve outcome in these patients.

## 1. Background

Liver cancer is currently estimated to be the sixth most commonly diagnosed cancer and the fourth leading cause of cancer-related deaths worldwide, accounting for 841,000 new cases and 782,000 deaths annually [1]. Hepatocellular carcinoma (HCC) is the most common primary liver tumor, with a rising incidence over the past decades in various populations [2,3]. Despite continuous advances in the management of chronic liver disease, in tumor detection and oncological treatment, the prognosis of HCC remains inferior in comparison to other tumor entities [1]. Some 90% of all HCCs have a known underlying etiology, first and foremost, viral cirrhosis [4], but a major shift in the spectrum of etiology is predicted for the next decades, with a declining burden of chronic hepatitis C disease due to widespread introduction of modern direct-acting antiviral drugs and a rise of non-alcoholic fatty liver disease (NAFLD)-associated carcinomas related to the worldwide obesity epidemic [5]. Only a small fraction of patients presents with early stages of disease, where curative strategies, such as orthotopic liver transplantation (OLT), liver resection (LR) and ablative techniques can be employed. The majority of HCC diagnoses are made at tumor stages that are beyond curative treatment, with palliative care as the only remaining option [6].

Proposals for the classification of HCC range from entirely clinical and radiological approaches to histopathological and molecular classification systems [7,8]. In the Western world, the Barcelona Clinic Liver Cancer (BCLC) classification is the most widely used prognostic staging system for HCC. The BCLC system combines liver function, performance status of the patient and the extent of tumor spread and distinguishes 5 stages of disease (0, A–D) [9]. While the BCLC classification constitutes a milestone in clinical decision-making, nowadays, the traditional BCLC stage-based therapy boundaries have blurred; LR is applied in advanced HCC, trans-arterial therapies can aid bridging of early tumors, while new ablative strategies are also employed in the treatment of larger HCCs with good results [10,11]. The biology of HCC with underlying liver disease necessitates a multidisciplinary cooperation and individual patient evaluation to achieve maximal oncological radicality without significantly compromising liver function. Oftentimes, the clinical approach is shaped by the personal experience and specialty of the treating clinicians, leading to a significant variation and heterogeneity in treatment procedures and long-term outcomes [12]. Clinical decision can be especially complex in cases with localized but unresectable disease or impaired liver function, where additive or complimentary approaches have evolved over time and the choice between treatment modalities is not supported by direct comparative evidence. This review aims to illustrate the scope of state-of-the-art treatments and treatment combinations for HCC, with a strong clinical focus on emerging multimodal and multidisciplinary therapies.

## 2. Orthotopic Liver Transplantation

Today, HCC in cirrhosis constitutes approximately one third of waiting list indications for OLT in Europe [13]. HCC was suggested as an indication for OLT early in the pioneering era of organ transplantation as it prevents complications of liver disease progression, such as hepatic encephalopathy, ascites and gastrointestinal bleeding and averts HCC recurrence. The latter, in particular, substantiates the hypothesis of OLT being superior to LR or ablation in terms of long-term oncological outcome [14]. Initial discouraging results of OLT for HCC can largely be attributed to an overly liberal scope of indication [15]. Defining the eligibility criteria for transplantation as a single lesion smaller than 5 cm or 2–3 lesions no larger than 3 cm and no macrovascular invasion—the so-called Milan criteria—led to a dramatic improvement of posttransplant outcomes [16]. Nowadays, the Milan criteria constitute the benchmark of patient selection for OLT with excellent 5-year survival rates of 75% [17]. Generally, the survival probability after OLT can be modelled—as reflected in the predictive “Metroticket” concept—as a continuum of outcome, with the risk for recurrence and death rising with increasing tumor burden [18]. Several groups have therefore advocated a moderate expansion of the Milan criteria to allow more patients access to the only potentially curative treatment while compromising the survival results only slightly. As such, more liberal selection patterns, for example the Up-to-Seven (seven as the sum of size (in cm) and number of tumors) and University of California San Francisco (UCSF) (solitary tumor ≤ 6.5 cm, or ≤ 3 nodules with the largest lesion ≤ 4.5 cm and cumulative tumor diameter ≤ 8 cm) criteria have shown acceptable results [18,19,20]. All selection systems that are based on tumor size face the drawback of a possible inaccuracy of preoperative radiological staging, leading to a discrepancy between the radiological tumor size and the tumor size in explant pathology. Large studies report radiological inaccuracy in up to 20-30% of cases, both over-and underestimating tumor size [17].

Apart from tumor size and number, markers like tumor differentiation, cancer-related symptoms and serum alpha-fetoprotein (AFP) levels are predictors of long-term post-OLT survival and can assist patient selection [21]. As such, the authors of the extended Toronto criteria aimed at identifying a subgroup of patients whose tumor exceeds the size-limits defined by the Milan criteria but has a favorable biology. A biopsy of the largest HCC lesion was taken to rule out poor tumor differentiation. While the long-term results of this approach showed a statistically significant lower incidence of HCC recurrence in patients within Milan criteria than beyond Milan criteria but within the scope of Toronto, the survival rates of the latter patient cohort were acceptable (incidence of HCC recurrence 13.1% vs. 29.8% (*p* < 0.001), 10-year OS 60% vs. 50% (*p* = 0.07) within Milan vs. beyond Milan and within Toronto, respectively) [17]. Another predictor of post-transplant outcome is microvascular invasion (MVI), indicating an aggressive tumor biology. MVI is a strong predictor of tumor recurrence and inferior survival and is more frequent in patients exceeding the Milan criteria, showing a correlation with tumor size [18]. 

In recent years, organ shortage evolved as a major limitation of OLT and the imminent need for organ pool extension has only been partially met by the increased utilization of so called “marginal” or extended-criteria donor (ECD) allografts [22]. In this regard, a pressing concern is the potential disadvantage for wait-listed patients with benign indications for transplantation who are competing for the same organs.

Arguing that patients with HCC within Milan have a similar potential benefit as patients with non-malignant indications, the Milan criteria have been incorporated into most organ allocation policies. Since the immediate prognosis of HCC patients is determined predominantly by the oncological disease, rather than by their liver function impairment, the Model for End-stage Liver Disease (MELD) score, which is reflective of liver function and constitutes the standard in most organ allocation systems, does not appropriately mirror the medical urgency of transplant candidates with HCC. In the United States and Europe, exceptional MELD points can therefore be assigned to United Network for Organ Sharing (UNOS) T2 Stage II HCC (single tumor 2–5 cm or 2–3 tumors under 3 cm), unless the lab-MELD score exceeds this number [23]. 

Furthermore, living donor liver transplantation (LDLT) can be offered to patients beyond Milan criteria to enable them to undergo transplantation or to patients within Milan criteria to shorten their waiting time. While LDLT enables clinicians to offer a potentially life-saving procedure, a higher rate of recurrence than after deceased donation, following an adjustment for tumor characteristics, was associated with LDLT in various Western series [24]. Even though OS between the two groups was similar, this phenomenon warrants further investigation. Possible hypotheses include the negative selection of tumor biology by “fast-tracking” the patient to transplantation without previous waiting times or bridging, and a regenerative stimulus of partial liver transplantation that may have a stimulatory effect on residual tumor cells [24]. 

Liver transplantation affords only a moderate survival benefit for patients with early-stage HCC and Child-Pugh A cirrhosis in comparison to LR or ablative treatments, suggesting that OLT for Child A cirrhosis with early-stage HCC does not make appropriate use of the scarce liver allograft pool and that patients with preserved liver function and early HCC are treated adequately with LR or ablation (Table 1) [25].

## 3. Liver Resection

**Figure 1:**
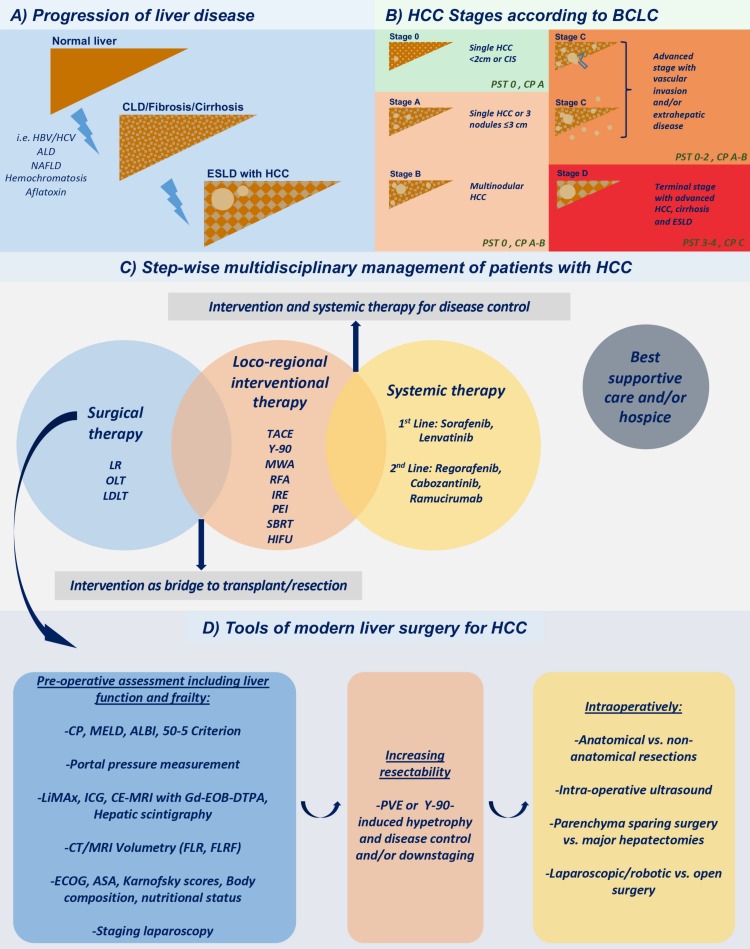
Multidisciplinary HCC evaluation and treatment. (**A**) Progression of liver disease; (**B**) HCC stages according to BCLC; (**C**) Step-wise multidisciplinary management of patients with HCC; (**D**) Tools of modern liver surgery for HCC.

Long-term survival rates after LR are only marginally inferior to the ones after transplantation, and are usually determined by intrahepatic tumor recurrence, which occurs in half of the cases at 3 years after liver resection or ablation [30,31]. Reasoning that LR, if technically attainable, reduces the stress on the limited donor organ pool and does not require long waiting times with potential treatment dropout, surgical resection is nowadays often performed in selected patients eligible for transplantation, reserving OLT for HCC patients whose severely impaired liver function precludes resection. While in most hepatic malignancies the attainability of curative resection depends predominantly on anatomic considerations and the percentage of tumorous tissue, HCC usually arises against the background of chronic liver disease. Resection in these high-risk cohorts is associated with an elevated risk of post-hepatectomy liver failure [32], and therefore necessitates detailed preoperative planning beyond a purely mechanistic approach as well as the implementation of modern parenchyma-sparing and less invasive surgical strategies (Figure 1).

While the original BCLC classification only proposed liver resection for BCLC stages 0 and A and defined other stages as “non-surgical”, today, the indication for surgery can be partially expanded to more advanced stages due to innovations and advances in surgical technique and perioperative management [9,33]. 

Preoperative assessment of portal hypertension (portal pressure measurement, splenomegaly, esophageal varices, platelet count), the prediction of the volume of the future liver remnant (FLR) or future liver remnant function (FLRF) as a percentage of the total liver volume, calculated from computed tomography (CT) or Magnetic Resonance Imaging (MRI) and metabolic liver-function tests, determination of Child-Pugh Score and even staging laparoscopy aid the evaluation of the underlying parenchymal disease. Liver volume, as a surrogate parameter of liver function, can be employed as a cutoff to determine the maximally resectable percentage of liver tissue. Although an FLR of ≥ 20% is a feasible threshold in a healthy liver, a pre-damaged fibrotic or cirrhotic liver necessitates an FLR of at least 40% [34]. In cases requiring extended resections, portal vein embolization (PVE) is a frequently used strategy to induce growth of the contralateral hemi-liver while causing atrophy of the ipsilateral lobe [35,36]. Although the effect of PVE is less pronounced in cirrhotic patients, the concept can expand the indication for surgery to select HCC cases with a relatively preserved liver function and an initially inadequate FLR as well as reduce the rate of postoperative complications [37,38]. 

Several factors which have previously been considered as absolute contraindications for surgery, such as portal hypertension, multifocal disease or isolated hyperbilirubinemia do not preclude successful resection today [28,39]. As such, a retrospective evaluation of the Italian Liver Cancer database (ITA.LI.CA.) suggested that impaired liver function (Child B, >MELD 9) and low performance status weigh more heavily in predicting the survival benefit of resection than strict adherence to BCLC staging. The authors concluded that in BCLC B and C patients, resection may also offer survival benefits over locoregional therapies, and should therefore be preferred whenever technically feasible [28]. Similarly, a randomized controlled trial (RCT) conducted in an Asian population demonstrated that in multifocal HCC outside the Milan Criteria, LR shows a clear superiority over trans-arterial chemoembolization (TACE) in terms of overall survival (OS) [40]. Although still controversial, portal vein tumor thrombosis, if involving only a segmental or second-order branch, does not prohibit surgery. Palliative strategies in this setting, for example TACE and Sorafenib, have resulted in inferior survival than surgery [41]. Even in selected cases of tumor invasion into the first-order branch of the portal vein or into the main portal trunk, LR with tumor thrombectomy can provide a survival benefit in comparison to palliation [42].

Anatomic LR encompasses the *en-bloc* removal of a liver segment nourished by a branch of the portal vein and hepatic artery. Non-anatomic or wedge resections spare a larger margin of liver parenchyma, which on the one hand, allows the preservation of a larger liver remnant with vital significance in patients with an impaired liver function, but, on the other hand, does not remove the potentially present intrahepatic satellite micro-metastases, resulting in an inferior oncological outcome [43]. 

As reported by a systematic review and meta-analysis, laparoscopic resection achieves similar survival results as conventional resection, with a reduced blood loss, a faster postoperative recovery and a lower rate of postoperative complications [44]. This observation has been confirmed in patients with cirrhosis, where a laparoscopic approach reduced the risk of post-hepatectomy liver failure [45,46]. Fewer data are available regarding laparoscopic major liver resection in HCC, but a small Japanese cohort exhibited shorter in-hospital stay and a reduced morbidity with the use of a minimally invasive approach [47]. However, no high-quality evidence from RCTs comparing laparoscopic versus open liver resection for HCC has been published yet [46]. It should be added that laparoscopic resection is usually performed in centers with extensive experience in laparoscopic and hepatobiliary surgery, necessitating a cautious interpretation of technical endpoints like conversion rate, operative time and blood loss. A further technique currently under investigation in highly specialized centers is minimal-invasive robotic surgery in HCC patients, which will be evaluated in future studies. 

## 4. Interventional Treatment

Locoregional therapies for HCC include a broad spectrum of techniques such as TACE, trans-arterial radioembolization with Yttrium-90 (Y-90), radiofrequency (RFA), microwave (MWA) and cryoablation, irreversible electroporation (IRE), percutaneous ethanol injection (PEI), high-intensity focused ultrasound (HIFU) and stereotactic body radiotherapy (SBRT) (Table 2). Locoregional therapies are nowadays applied for a wide range of curative and palliative indications, including their repeat application, their combination with resection, bridging the waiting time to transplantation and down-staging advanced tumors to fulfil criteria of resection or transplantation. Furthermore, even BCLC stage C patients with low extrahepatic tumor burden may benefit from locoregional therapies to slow hepatic tumor progression, which constitutes their leading cause of death [48]. 

Today’s available evidence on locoregional therapies is partially limited due to the novelty of some of the techniques, such as IRE and SBRT. A paucity of RCTs impairs the direct comparability of locoregional techniques in specific disease stages and settings. 

### 4.1. Ablative Techniques

Tumor ablation employs either thermal or non-thermal techniques to destroy malignant cells and a narrow ablative margin of 0.5–1 cm of non-malignant parenchyma, thus sparing more surrounding tissue than after resection [49]. Chemical ablation (PEI or acetic acid injection) is a generally well-tolerated technique, even in patients with liver cirrhosis, and has the highest efficacy in HCC < 2 cm [50]. However, as it is associated with higher recurrence rates and inferior survival in comparison to hyperthermic ablation [51,52], it only plays a subordinate role in HCC treatment today, having largely been replaced by more modern techniques. Hyperthermic ablation includes radiofrequency and microwave techniques and subjects the surrounding tissue to cytotoxic temperatures, causing coagulation necrosis [51].

The most widely employed ablative technique is RFA, the principle of which relies on ion agitation and heat generated due to the electrical impedance of the tissue (Joule effect) [49]. The RFA treatment yields the best results in HCCs smaller than 2 cm, where sustained, local complete radiological response rates of 97.2% after a median follow-up of 31 months have been documented in patients with Child A cirrhosis [52]. In this cohort, RFA affords a good local control with a local recurrence rate around 5% [53]. The efficacy of RFA diminishes with increasing tumor size, and repeat interventions become necessary to encompass the target volume [49]. 

In patients with BCLC 0 and A tumors, RFA is a valid treatment strategy, especially if the patient is not eligible for surgical resection. In clinical decision-making, the choice of treatment in small HCCs is often coined predominantly by the tumor localization. In small, centrally situated tumors, RFA offers a tissue-sparing approach, but tumor proximity to large vessels impairs RFA efficacy due to the cooling effect of blood flow (heat sink effect) [54,55]. Similarly, ablation of subcapsular lesions, or of lesions in proximity to the gall bladder or the diaphragm carry a higher risk of complications and tumor recurrence [56]. While the peri-interventional outcome of both LR and RFA is strongly determined by liver function and tumor size, the latter weighs more heavily in RFA, while liver function is a stronger predictor in resection [57]. A Cochrane systematic review concluded from four RCTs comparing RFA to LR in early HCC that LR has a significantly lower cancer-related mortality, whereas the rate of serious adverse events is lower when RFA was performed. No difference in all-cause mortality was observed between the two treatment options [58]. 

In MWA, electromagnetic microwaves cause frequency-dependent flipping of water molecules due to their bipolar character, generating consistent intra-tumoral heat [59]. While the clinical evidence available on this modality is insufficient to support a clear recommendation, an RCT from Japan concluded that MWA may yield similar results as RFA [60]. Possible advantages over RFA may lie in the lesser susceptibility of MWA to heat sink effects of the hepatic vasculature, as well as in the feasibility of ablating larger tumor volumes [61]. According to a single-center experience published by Liu et al., ablation rates of around 95% can be achieved in lesions measuring up to 5 cm, while the complete ablation rate in tumors exceeding 5 cm is significantly lower at 75% [62].

IRE is a relatively new ablative technique that employs high-frequency electric pulses to induce transmembrane pores and thus leads to cell death while sparing the extracellular matrix. IRE is especially effective in tissues with a high cell density. The non-thermal nature of IRE seems to be a significant advantage in proximity to blood vessels and heat-sensitive structures such as large bile ducts [51]. While IRE provides an excellent local control of the ablation zone, recurrence due to needle tract seeding may be a possible disadvantage of IRE and has been documented in up to a quarter of treated cases [63]. A similar problem was initially encountered in RFA, until the introduction of needle tract ablation lowered the incidence to 0.5% [64]. This illustrates the need for technical optimization of this method, along with the necessity to investigate the potential and risks of IRE beyond the currently available studies.

### 4.2. TACE

Almost 20 years after the Barcelona group, led by Jordi Bruix, pioneered the BCLC classification and suggested TACE in palliative intent for intermediate-stage (BCLC B) patients (localized disease, exceeding 3 nodules 3 cm in diameter, Eastern Co-operative of Oncology Group (ECOG) performance status 0), current clinical guidelines (i.e., EASL) still recognize and endorse this recommendation [9,13]. The principle of TACE relies on targeting the arterial hypervascularization of HCC. Tumor necrosis is achieved by embolization of the arterial blood supply with either a suspension of lipiodol and a chemotherapeutic agent and gelatin sponge or with drug-eluding beads loaded with doxorubicin. While TACE results in an inferior OS than LR in resectable lesions, several RCTs have provided evidence of an improved survival after TACE compared to best supportive care in BCLC B patients beyond resection criteria, making TACE an integral part of treatment in this cohort [40,65,66]. The heterogeneity of the BCLC B group, for example, concerning liver function (Child-Pugh class A or B) and varying tumor burden (multinodular and/or large HCC), necessitates a careful patient selection for TACE and may lead to a significant inconsistency and bias in available studies [67]. Consensus exists that, irrespective of specific laboratory thresholds which vary significantly between investigators, special attention must be given to an adequate hepatic function, owing to the elevated risk of acute liver failure after TACE. This risk is significantly increased in patients with Child-Pugh B cirrhosis, while Child-Pugh class C liver function is predominantly viewed as a contraindication for TACE [68,69]. Nevertheless, super-selective TACE conducted in the most peripheral accessible feeding artery and thus embolizing nearly no healthy tissue may be a feasible alternative in Child C patients that affords a survival benefit in comparison to best supportive care, but limited evidence, patient cohort heterogeneity and a variation between interventional techniques preclude its incorporation into current clinical guidelines [70]. Furthermore, TACE is not indicated in patients with central portal vein thrombosis without collateralization, since TACE may further compromise the hepatic blood flow and cause an acute deterioration of liver function [71]. 

### 4.3. Y-90

Radioembolization is most commonly performed with Y-90-coated glass- or resin microspheres. Y-90 is a β-radiation-emitting isotope. The particles are delivered via a microcatheter placed in the target liver artery, with the small particle size allowing for a penetration and dispersion in the tumor while maintaining a sufficient residual blood flow in the vessel [72]. To prevent extrahepatic microsphere administration, coil-embolization of hepatic artery branches is usually performed prior to the intervention. Furthermore, a macroaggregated albumin perfusion scan (MAA scan) can rule out possible non-target embolization and ensure a sufficiently low lung-shunt-fraction (<5%). Y-90 can be performed across the stages BCLC A-C and even selectively in BCLC D patients and compares favorably with the reported survival expectations, but the evidence in this patient cohort is limited [73]. Lobar portal vein thrombosis does not preclude Y-90 with resin microspheres, as it confers a less dominant effect on blood flow dynamics than TACE, and has been confirmed as an effective strategy in this patient collective [74]. It should be noted that two recent phase III trials from an Asian-Pacific, and from a Western cohort failed to prove a superiority of Y-90 over Sorafenib in locally advanced and inoperable BCLC C HCC, reporting no significant difference in OS and progression-free survival (PFS). An interesting finding of both studies was that Y-90 resulted in a lower incidence of hepatic tumor progression and had a favorable toxicity profile when compared to Sorafenib [75,76].

### 4.4. Further Innovative Locoregional Approaches

The application of high intensity ultrasound energy leads both to thermal ablation, as well as to non-thermal effects in the context of cavitation and mechanical tissue disruption due to boiling bubbles. While HIFU has mostly been used in the treatment of prostate cancer and uterine fibroids, a small number of trials has also evaluated HIFU for HCC [77]. Still in its infancy for the treatment of HCC, it seems that the technique can be applied in proximity to large vessels, where the rate of complete ablation is around 50% [78]. Tumor localization can limit the therapeutic window because ablation in unfavorable localizations is associated with an increased rate of side effects, namely, thermal damage [77].

Radiotherapy of the liver is limited by the risk of radiation-induced liver disease. SBRT, an external beam radiation therapy, has gained recognition as a strategy to deliver radiation focally and to therefore reduce the risk of liver toxicity. Still, Child-Pugh B patients are more prone to experience an increased liver toxicity already after low radiation doses [79]. Nevertheless, excellent local control around 90% at 1 year which, for tumors larger than 2 cm exceeds the results of RFA, can be achieved with SBRT [79,80]. Today, SBRT is mostly applied in HCC > 4 cm if the location of the lesion prohibits thermal ablation and can be considered for HCC recurrence after ablation [81]. However, a randomized, prospective comparison of SBRT with other locoregional therapies in different settings is needed to fully determine the future value of this technique in HCC treatment.

## 5. Systemic Treatment

Not only is HCC a highly chemotherapy-resistant tumor, but the applicability of most chemotherapy regimens is severely limited by the underlying liver disease. Therefore, for a very long time, no systemic standard of care was available for patients with advanced HCC [92,93]. The most prevalent driver mutations in HCC affect the TERT promoter, TP53 and the Wnt/β-catenin signaling pathway and are not yet amenable to routine therapeutic targeting. At the same time, molecular alterations that constitute established therapeutic targets in other tumor entities could only be identified in a small fraction of HCC patients [94]. First reports of the multi-kinase inhibitor Sorafenib (Nexavar®) in 2006 were considered a milestone in HCC treatment, because, for the first time, a systemic therapy showed some survival benefit in advanced HCC [95]. Sorafenib targets the Raf–MEK–ERK pathway as well as several receptor tyrosine kinases, including vascular endothelial growth factor receptor (VEGFR) 2 and 3, platelet-derived growth factor receptor (PDGFR), Ret, FLT3, and c-Kit [96,97,98]. The SHARP trial was the first phase III RCT to compare Sorafenib with placebo in 602 patients with advanced HCC and mostly (97%) Child-Pugh class A. Significantly increased OS and PFS under Sorafenib treatment compared to placebo (10.7 vs. 7.9 months and 5.5 vs. 2.7 months, respectively) were observed [99]. The data were validated in an Asian cohort, where the sorafenib group had significantly longer OS and prolonged time to progression (TTP) (6.5 vs. 4.2 months, and 2.8 vs. 1.4 months, respectively) [100]. As a result of these RCTs, Sorafenib is nowadays considered as the mainstay of palliative treatment in BCLC C patients (extrahepatic spread, macrovascular invasion, cancer-related symptoms) [13]. The most frequent grade 3/4 drug-related adverse events of sorafenib include hand-foot skin reaction, diarrhea and fatigue [100]. However, the median OS improvement of 2.8 months and 2.3 months in the Western and Asian-Pacific populations, respectively [99,100], can be regarded as unsatisfactory and illustrates the need for further clinical strategies. Yet, for almost a decade, several subsequent randomized phase III trials with new molecular agents failed to either prove non-inferiority or to surpass the outcome achieved with Sorafenib [94]. 

In the first line setting, a phase III trial confirmed the non-inferiority of the multi-kinase inhibitor Lenvatinib in comparison to Sorafenib, with a median survival of 13.6 months in the Lenvatinib group and 12.3 months in the Sorafenib group, leading to FDA approval for unresectable HCC in 2018 [101,102]. The PD-1 inhibitor Nivolumab may significantly shape future treatment perspectives for advanced HCC. A phase I/II trial (CheckMate 040) for this checkpoint inhibitor showed encouraging response rates of 15–20% (compared to 2–3% on first-line Sorafenib [99,100]) [103]. Data from the randomized phases of this trial comparing Nivolumab with Sorafenib in the first-line setting are eagerly anticipated. 

**Figure 2:**
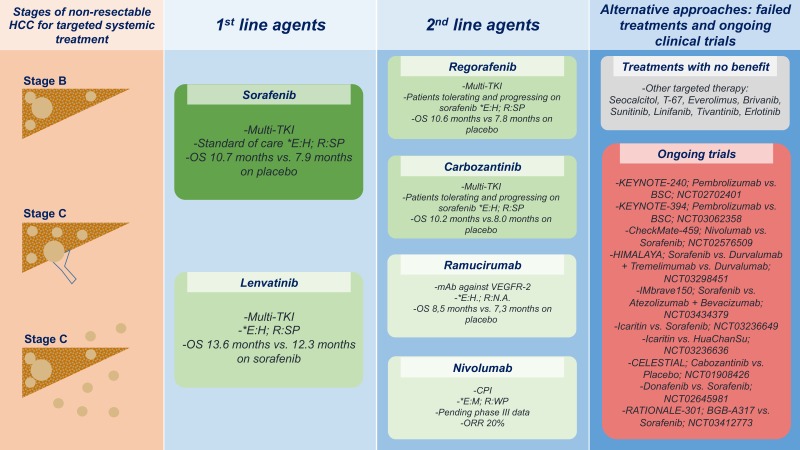
Systemic targeted therapy options for intermediate and advanced HCC. BCLC Stage B (multinodular) and Stage C (portal vein invasion or extrahepatic disease). Abbreviations: BSC—best supportive care, CPI—checkpoint inhibitor, E:H—evidence high; E:M—evidence medium, mAb—monoclonal Antibody, ORR—objective response rate, R:NA—recommendation: not available, R:SP—recommendation: strong positive, R:WP—recommendation: weak positive, TKI—tyrosine kinase inhibitor, VEGFR—vascular endothelial growth factor receptor. Recommendations derived from: European Association for the Study of the Liver, Hepatology, 2018 [13].

Only recently, two agents have shown survival benefits in the second-line setting. The multikinase inhibitor Regorafenib was shown to increase OS in patients with disease progression on Sorafenib In the RESORCE trial [104]. In 2018, the results from a phase III trial comparing the MET, VEGFR 1,2,3 and AXL inhibitor Cabozantinib with placebo in the second-line setting following Sorafenib treatment were published. Significantly improved OS and PFS in the Cabozantinib group (10.2 vs. 8.0 months and 5.2 vs. 1.9 months, respectively) in comparison to the placebo were reported [105] (Figure 2). Further targeted systemic therapy approaches including agents that failed to show a benefit as well as currently running trials are depicted in Figure 2. 

## 6. Multimodal Strategies

### 6.1. Bridging to Transplant

Locoregional therapies have gained recognition as a method to achieve local tumor control and therefore reduce wait-list drop-out and recurrence after transplantation. An analysis of the Scientific Registry of Transplant Recipients from the United States showed that patients who received pre-transplant treatments, mostly TACE and RFA, had a superior adjusted 3-year post-transplant survival than those who did not [106]. 

No conclusive evidence on the superiority of a single technique as a bridge to transplantation has yet been brought forward. In most published series, the study populations received heterogenous treatment modalities prior to transplantation, often depending on institutional preference [106]. As bridging treatments are employed in different contexts and have different contraindications, the comparability between modalities, especially in form of an RCT, is limited. The feasibility and efficacy of TACE, RFA and SBRT as bridging therapies have been confirmed in terms of the reduction of wait-list dropout rates and improved post-transplant survival rates [107,108]. A non-randomized comparative analysis of bridging with TACE vs. bridging with Y-90 reported a significantly shorter median time to overall progression in the TACE cohort (12.8 months) than in the Y-90 cohort (33.3 months), but dropout rates, postoperative complications and 5-year OS were similar between the groups [109]. 

### 6.2. Tumor Downstaging

It is postulated that tumors with a favorable biology are more prone to respond to locoregional therapies and have an improved recurrence-free survival after transplantation. Tumor downstaging is defined as the utilization of locoregional or systemic therapies to decrease the size of HCC lesions to meet current selection criteria for OLT and is considered successful if the tumor remains stable after a follow-up period of at least three months [110,111]. Several sources have suggested that in patients with tumor mass beyond Milan criteria, tumor size reduction and downstaging to Milan prior to transplantation results in an excellent post-transplantation outcome [112]. Therefore, patients with a tumor downstaged into Milan criteria gain exceptional points on the UNOS waiting list similarly to patients within Milan.

### 6.3. Salvage Liver Transplantation

Salvage OLT has been proposed for patients with HCC recurrence or deteriorating liver function after LR or loco-regional treatment. Arguing that in the majority of recurrences, the patients present within criteria for transplantation, several working groups suggested that salvage OLT may allow for a more effective management of liver allografts while reducing the number of patients at risk of wait-list drop-out [38,113]. While the indications and feasibility of salvage OLT are still subjects of an ongoing debate, mainly due to the concerns of a higher rate of post-OLT recurrence, a meta-analysis showed that salvage OLT yields similar surgical and short- and long-term oncological outcomes as upfront OLT [114]. However, due to the complexity of the interplay between oncological, surgical and donor-mediated factors, multicentric RCTs are still needed.

### 6.4. Locoregional Strategies and LR

Just as tumor size reduction using locoregional therapies can enable a patient to undergo OLT, it can also aid surgical resection in patients with compromised liver function. Not only does Y-90 reduce tumor size, but it also leads to a compensatory hypertrophy of the contralateral lobe, probably due to the irradiation of the non-tumorous liver parenchyma and a subsequently constrained liver function of the radio-embolized lobe [115]. This Y-90-induced atrophy-hypertrophy sequence is of special interest in functionally irresectable HCC patients with tumor burden confined to the right hepatic lobe, where hypertrophy of the left hemiliver may result in reaching secondary resectability criteria. Thus, the combination of local control and hepatic volume changes afforded by Y-90 can facilitate surgical resection of previously irresectable tumors as part of a two-stage approach. Nevertheless, since PVE affords faster and more significant liver growth, it is considered the gold standard in patients whose hepatic reserve is the only surgical concern. Y-90 may benefit those patients in which additional local tumor spread precludes primary resection [116]. A concept only investigated in small, single-center case series is sequential preoperative TACE and PVE. Besides an increase in the rate of FLR hypertrophy, probably due to the occlusion of arterio-portal shunts, an additional anti-tumorous effect of this strategy is suspected [117]. Furthermore, the controversial significance of an accelerated hypertrophy to increase resectability rates induced by the “Associating Liver Partition and Portal Vein Ligation for Staged Hepatectomy” (ALPPS) approach in HCC patients has been explored in small and highly selected cohorts [118,119].

### 6.5. Synergistic Effects of Locoregional Therapies

The limited efficacy of RFA for HCCs larger than 3 cm has led to the hypothesis that a combination of RFA with TACE may improve the results in these tumors by limiting the arterial hepatic blood flow and thus perfusion-mediated tissue cooling. Indeed, the combination with TACE in tumors with a maximum diameter of 3-5 cm results in an extension of the ablated area, reduces the number of required interventions and slows local tumor progression [120]. However, in patients eligible for surgical resection, TACE followed by RFA does not attain the oncological and survival results of LR [121]. 

Another promising approach to enhance the ablated tumor volume is combining RFA with the infusion of the heat-activated agent lyso-thermosensitive liposomal doxorubicin (LTLD). LTLD is activated at a temperature threshold of ≥ 39.5 °C and, by increasing the probability of thermally mediated tissue necrosis, leads to a synergistic effect when combined with RFA [122]. 

### 6.6. Adjuvant therapy after LR, ablation, TACE and OLT

In the face of the high probability of tumor recurrence in the cirrhotic liver, adjuvant treatment after liver-directed procedures to reduce this dysplastic potential seems like a reasonable suggestion [30,31,123]. However, the adjuvant application of Sorafenib after LR or ablation did not lead to an improved median recurrence-free survival in comparison to the placebo in previous reports [124]. 

The role of combined systemic and liver-directed treatments in inoperable intermediate- or advanced-stage HCC is yet to be determined. The combination of TACE with antiangiogenetic therapies was suggested as a means of optimizing disease control and reducing recurrence after TACE. A possible explanation for tumor recurrence after TACE is that TACE induces hypoxia, leading to the induction of tumor angiogenesis via upregulation of vascular endothelial growth factor (VEGF) [125]. While the safety and feasibility of targeting the vascular tumor supply by combining TACE and Sorafenib were proven in the phase II SPACE trial, both groups had a similar time to progression (TTP) and the Sorafenib group had a non-significantly longer median time to macrovascular invasion and extrahepatic spread [126]. Concurrent Sorafenib and TACE did not improve PFS in comparison to TACE plus placebo in an RCT of patients with unresectable HCC (TACE 2) [127]. To date, the only RCT meeting the primary endpoint PFS in this setting (13.5 vs. 25.2 months in TACE and Sorafenib vs. TACE and placebo (*p* = 0.006)) is the TACTICS trial, which was characterized by lower Sorafenib dosage and longer administration times (400 mg/day prior to TACE, then 800 mg/day during and after TACE) and was conducted in an Asian cohort [128]. The disparity in the results of these trials warrants further clinical investigation. The results of the SORAMIC trial evaluating the benefit of adding Y-90 to sorafenib treatment in patients not eligible for surgery or TACE are eagerly anticipated. A preliminary safety analysis of the first 40 patients showed a similar incidence of total and grade ≥3 adverse events [129]. Furthermore, a currently accruing RCT evaluates SBRT combined with Sorafenib versus Sorafenib alone (NCT01730937) [130].

Adjuvant chemotherapy after transplantation was initially suggested to lower recurrence rates by eliminating tumor cells which are potentially disseminated during manipulation of the liver as well as controlling micro-metastases. While small historical series showed a survival benefit of adjuvant chemotherapy, the applicability of these series is limited, since they included many patients with large tumors that are beyond today’s selection criteria for OLT [131]. More recent investigations of adjuvant chemotherapy, such as low-dose doxorubicin, showed no survival benefit of an adjuvant therapy, which is in line with the low chemo-sensitivity of HCC [132].

## 7. Future Perspectives and Remaining Challenges

The treatment of HCC is demanding and complex, as the tumor arises at the intersection of several pathologies: an underlying chronic pathology of the liver which limits therapeutic possibilities and has a significant residual oncogenic potential as well as the tumor biology itself. Therefore, the necessity of weighing individual factors, factors of tumor biology and frontline clinical developments makes a multidisciplinary patient evaluation indispensable [133].

These developments towards personalized cancer care may eventually be augmented by the study of the molecular landscape of HCC, which has not yet shaped clinical practice. For example, no predictive value of the molecular HCC subclasses for Sorafenib sensitivity has been noted yet [94]. In the future, the search for mutated oncogenes may identify tumor subgroups susceptible to molecular targeting, such as the post-hoc identification of a longer TTP under Tivantinib in patients with MET-high tumors [134]. Thus, biomarker-embedded clinical trials conducted in specific patient populations may further shape the future of a personalized HCC treatment [94].

Obtaining predictors of outcome and recurrence biology is a relevant endeavor in all malignant tumors, but especially so in HCC, where this may support the intense ethical debate of indications for OLT. The optimization of HCC patient selection to release the pressure on waiting lists may be achieved by advancing onco-surgical strategies, by a broadened indication for salvage liver transplantation and by LDLT. New technical developments in liver transplantation may expand the donor pool to previously discarded organs [22,29].

In the future, a spectral change of tumor etiology from viral cirrhosis due to Hepatitis B and C virus infection to NAFLD can be predicted. This is caused, on the one hand, by the widespread implementation of Hepatitis B vaccines and the success of direct antiviral hepatitis C treatments, and, on the other hand, by the dramatic epidemic of obesity and metabolic syndrome on a global scale [135]. These patients are characterized not only by the lack of a classical cirrhosis but also present with metabolic syndrome, major comorbidities such as severe obesity, diabetes mellitus type 2, chronical kidney disease and coronary heart disease [136]. Since the current scope of evidence is predominantly based on studies conducted in patient cohorts with viral and alcoholic tumor etiology and cirrhosis, the above-mentioned populational developments will challenge current therapeutic guidelines and classifications and necessitate a reevaluation of our treatment strategies in the future.

In conclusion, significant advances have been made in the study and development of surgical, loco-regional and systemic treatment modalities for HCC. Nevertheless, in the face of the global disease burden and the limited survival in advanced stages, further research is fundamental to improve the prognosis of patients with HCC (Table 3). The multitude of available complimentary and additive treatment modalities should encourage clinicians to implement a multidisciplinary treatment approach to improve the outcome in these patients.

## Figures and Tables

**Table 1:** Clinical decision-making: liver transplantation vs. resection in BCLC 0 and A (and B).

Modality	Liver Transplantation		Liver Resection
	DDLT	LDLT	
**Indications**	HCC within Milan criteria or down-staged into Milan	HCC within Milan and reduction of waiting time HCC beyond Milan (no exceptional MELD points in organ allocation)	Preserved function (CP A, MELD ≤9) and sufficient FLR; Individual evaluation in cases of vascular invasion
**Advantages**	Cures underlying liver disease Feasible and beneficial in advanced cirrhosis	No wait-list drop-out, releases pressure on waiting list Potentially curative beyond Milan	Feasible in large tumors >5 cm [26] Releases pressure on waiting list
**Disadvantages**	Progression on waiting list, wait-list drop-out Allograft shortage Life-long immunosuppression	Higher rate of recurrence due to “fast-tracking” [27]/regeneration [24] Need for a suitable donor; donor morbidity	Inferior recurrence-free survival Progression of liver disease despite LR High-risk in patients with CP B/MELD >9 cirrhosis [28]
**Strategies for optimization**	➔ Bridging strategies ➔ ECD and organ reconditioning to expand donor pool [29]	➔ Bridging to aid patient selection and observe tumor biology [24]	➔ Rescue OLT ➔ Previous PVE, Y-90

Abbreviations: CP—Child-Pugh, DDLT—deceased donor liver transplantation, ECD—extended-criteria donor, FLR—future liver remnant, HCC—hepatocellular carcinoma, LDLT—living donor liver transplantation, LR—liver resection, MELD—Model for End-stage Liver Disease, MVI—Macrovascular invasion, OLT—orthotopic liver transplantation, PVE—portal vein embolization, Y-90—trans-arterial radioembolization with Yttrium-90.

**Table 2:** Overview of loco-regional treatment strategies for HCC.

Modality	Technique	Indication	+	−
**RFA**	ablative; thermal, Joule effect [49]	BCLC 0, A, B tumor < 2–3 cm not subcapsular/perivascular/adjacent to gallbladder/diaphragm	lower rate of serious adverse events than LR [58] tissue-sparing [49] most extensively studied ablation technique, broad clinical experience [82]	reduced efficiency when HCC is subcapsular/perivascular/adjacent to gallbladder/diaphragm [56], />3 cm higher cancer-related mortality than LR [58]
**MWA**	ablative; thermal, agitation of water molecules and friction	BCLC 0, A, B Similar profile to RFA tumor ≤ 5 cm	less heat sink effect and shorter duration of therapy than RFA [51] efficient in tumor volumes ≤ 5 cm [61]	reduced efficacy in tumors >5 cm [62] treatment effect varies between different devices [83]
**IRE**	ablative, non-thermal, electric pulses create irreparable membrane pores that cause breakdown of transmembrane potential	perivascular locations [51] applicable in peribiliary locations (limited evidence) [63]	no heat sink effect applicable in perivascular locations [51] preservation of the extracellular matrix increased effect in tissues with high cellularity (e.g., tumors)	elevated incidence of needle tract seeding [63] insertion of several needles necessary [63] limited evidence and experience requires general anesthesia [13]
**TACE**	chemoembolization with doxorubicin or cisplatin (conventional TACE or with drug-eluting beads)	palliative indication BCLC B, CP A (CP B) [65,66] subsegmental TACE: Very selectively in CP B (and superselective TACE rarely in CP C) [70,84] higher incidence of post-embolization syndrome adjacent to gallbladder [85]	extensively studied, safety proven [84]	local tumor recurrence higher than after LR/RFA [84] elevated risk of liver failure in cases with CP B, C [68,69] and portal vein thrombosis [71] post-embolization syndrome [85], decreased by dexamethasone [86]
**Y-90**	arterial application of Yttrium-90 [72]	BCLC A-C (D) [73]	applicable in presence of PV thrombosis [74] favorable toxicity in comparison to Sorafenib [75]	less clinical experience than with TACE
**SBRT**	high radiation doses delivered in few fractions	CP A (and B) [79]	excellent local control applicable to large tumors [87]	elevated risk of liver toxicity in CP B [79]
**PEI**	ethanol injection causes coagulation necrosis	only limited role in HCC treatment today highest efficacy in HCC <2 cm [50]	moderate cost, simple, attractive for developing regions [49] feasible in cirrhosis [49] well-tolerated [50]	heterogenous intra-tumoral distribution, especially in the presence of septa [50] higher recurrence and inferior survival than ablation [58,88] multiple injections necessary [51] obsolete technique
**HIFU**	ultrasound; thermal (due to absorption of energy) and non-thermal (cavitation, boiling bubbles) effects [77]	largely experimental, investigated as bridge to transplant in CP cirrhosis [89]	selective; ablation in proximity to large vessels feasible [78] non-invasive combination of HIFU and TACE may be a more effective option than TACE monotherapy for HCC < 5 cm [90]	limited inter-costal therapeutic windows can cause reflection and unintended burns [77] Future: intrapleural fluid infusion? [91] significant treatment disruption by breathing motion—often requires mechanical ventilation

**Table 3:** Future perspectives in basic and applied research in HCC treatment.

Direction of Research	Specifications	References
**Loco-regional therapies**	Optimizing ablation strategies (laser ablation, cryoablation, radiosurgery, IRE technologies reducing the risk of seeding metastases)	[137,138]
**OLT and LR**	Better utilization of ECD allografts and LDLT to expand the donor pool Laparoscopic and robotic liver surgery Perioperative immuno-nutrition Trigger liver regeneration to increase an atrophy-hypertrophy complex and achieve resectability (i.e., ALPPS, PVE or subcellular manipulation of liver regeneration pathways)	[134,139,140]
**Systemic therapy/targeted therapy**	Biomarker-enriched clinical trials Pathway approach: TGF-β, FGFR, RAS, MET signaling Immune checkpoint inhibitors Novel agents with better tolerability and higher efficacy Reversal of multi-drug resistance in HCC cells Oncolytic virus therapies Novel chemotherapeutic approaches for HCC in non-cirrhotic patients Nanoparticle-mediated targeted drug delivery systems	[13,103,105,134,141,142,143]
**Tumor biology and Biomarkers**	Personalized approach to polyclonality, tumor heterogeneity and multicentricity Targeting the tumor microenvironment/stroma Targeting epigenetic modifiers (e.g., DNA methyltransferases or histone deacetylases) siRNAs/miRNAs Liquid biopsy: acquiring predictive biomarkers, tracing tumor dynamics and mutational drift, early detection	[94,134,144]
**Imaging techniques/Radiomics**	Radiomics: prognostic and predictive markers, e.g., preoperative estimation of recurrence Noninvasive surrogate markers of histological differentiation, microvascular invasion, molecular pathway upregulation Body composition and nutrition assessment as a potential underlying cause in NAFLD and HCC (i.e., myosteatosis and pro-inflammatory regulation)	[145,146,147]
**Multimodal approaches**	Antiviral therapy in combination with surgical and locoregional treatment External beam radiotherapy combined with TACE Electrochemotherapy (combined IRE and chemotherapy) Adjuvant therapy options	[148,149,150]

Abbreviations: DNA—deoxyribonucleic acid, FGFR—fibroblast growth factor receptor, MET—mesenchymal-epithelial transition factor, miRNA—micro ribonucleic acid, RAS—Rat sarcoma, siRNA—small interfering ribonucleic acid, TGF-β—Transforming growth factor beta.

## Abbreviations

AASLD	American Association for the Study of Liver Diseases
AFP	alpha-fetoprotein
ALBI	Albumin-Bilirubin Grade
ALD	alcoholic liver disease
ALPPS	Associating Liver Partition and Portal Vein Ligation for Staged Hepatectomy
ASA	American Society of Anesthesiologists
BCLC	Barcelona Clinic Liver Cancer
CE	contrast enhanced
CIS	Carcinoma in situ
CLD	chronical liver disease
CP	Child Pugh
CT	computed tomography
ECD	extended criteria donor
ECOG	Eastern Co-operative of Oncology Group
ESLD	end-stage liver disease
FLR	future liver remnant
FLRF	future liver remnant function
HBV	hepatitis B virus
HCC	hepatocellular carcinoma
HCV	hepatitis C virus
HIFU	high-intensity focused ultrasound
ICG	indocyanine green
IRE	irreversible electroporation
ITA.LI.CA.	Italian Liver Cancer database
LDLT	living donor liver transplantation
LiMAx	Liver Maximum Capacity Test
LR	liver resection
MELD	Model for End-stage Liver Disease
MRI	magnetic resonance imaging
MWA	microwave ablation
NAFLD	non-alcoholic fatty liver disease
OLT	orthotopic liver transplantation
OS	overall survival
PEI	percutaneous ethanol injection
PFS	progression-free survival
PST	performance status
PVE	portal vein embolization
RCT	randomized controlled trial
RFA	radiofrequency ablation
SBRT	stereotactic body radiotherapy
TACE	trans-arterial chemoembolization
TTP	time to progression
UCSF	University of California San Francisco
VEGF	vascular endothelial growth factor
VEGFR	vascular endothelial growth factor receptor
Y-90	trans-arterial radioembolization with Y-90
